# Modified albumin–bilirubin grade predicts overall and progression-free survival in metastatic gastric cancer

**DOI:** 10.17305/bb.2026.14188

**Published:** 2026-05-05

**Authors:** Fatih Sargin, Zeynep Gok Sargin, Ayse Karaduru Avci, Feyza Cetin Cansiz, Ismail Beypinar, Muslih Urun, Yusuf Ilhan, Selman Celebi

**Affiliations:** 1 Department of Intensive Care Medicine, Antalya City Hospital, Antalya, Türkiye; 2 Department of Gastroenterology and Hepatology, Health Sciences University, Antalya, Türkiye; 3 Department of Internal Medicine, Antalya City Hospital, Antalya, Türkiye; 4 Department of Medical Oncology, Alaaddin Keykubat University, Antalya, Türkiye; 5 Department of Medical Oncology, Van Yüzüncü Yıl University, Van, Türkiye; 6 Department of Medical Oncology, Antalya City Hospital, Antalya, Türkiye

**Keywords:** Gastric cancer, metastatic disease, modified albumin–bilirubin score, mALBI, survival

## Abstract

Gastric cancer (GC) ranks among the most prevalent and lethal cancers worldwide. The albumin–bilirubin (ALBI) score was initially developed to assess liver function in patients with hepatocellular carcinoma. Its modified version, the modified ALBI (mALBI) score, may offer prognostic insights for other malignancies. This study aims to evaluate the prognostic significance of mALBI grade in patients with metastatic GC. Between August 2021 and June 2025, a total of 93 patients with metastatic GC were included in this retrospective multicenter cohort study. mALBI scores were calculated based on serum albumin and total bilirubin concentrations. Patients were classified according to the original mALBI grading system into Grade 1, Grade 2a, Grade 2b, and Grade 3. Overall survival (OS) and progression-free survival (PFS) were assessed using the Kaplan–Meier method, while independent prognostic factors were analyzed via Cox regression. Kaplan–Meier analysis indicated significant differences in OS (χ^2^ ═ 44.156, *P <* 0.001) and PFS (χ^2^ ═ 22.142, *P < * 0.001) across the four mALBI grades. Utilizing established mALBI grade thresholds and prior binary grouping methods in GC, patients were further divided into low mALBI (Grades 1 and 2a) and high mALBI (Grades 2b and 3) groups. Those with high mALBI grades exhibited significantly worse median OS compared to patients with low mALBI grades (271 vs. 561 days, *P ═* 0.002), as well as significantly shorter median PFS (199 vs. 423 days, *P < * 0.001). In multivariate analysis, mALBI grade remained independently associated with both OS (*P < * 0.001) and PFS (*P ═* 0.001). Patients with lower mALBI grades demonstrated considerably better survival outcomes than those with higher grades. Due to its straightforward calculation from routine laboratory tests, mALBI grade may serve as a valuable prognostic marker for survival stratification in metastatic GC. Nonetheless, prospective validation in larger and more diverse cohorts is necessary before its integration into standard clinical practice. Furthermore, additional studies should explore whether interventions aimed at enhancing nutritional status and liver function can positively impact outcomes in high-risk patients.

## Introduction

Gastric cancer (GC) is one of the most prevalent and lethal cancers worldwide [1]. Despite significant advancements in therapeutic strategies, GC is frequently diagnosed at advanced stages, leading to poor prognoses and elevated recurrence rates [2]. Most deaths related to GC occur within the first year following diagnosis, underscoring the disease’s aggressive nature and the urgent need for effective prognostic grading tools [3, 4]. Traditionally, the prognostic assessment of GC has relied on pathological staging, with the American Joint Committee on Cancer (AJCC) tumor-node-metastasis (TNM) staging system being the most widely used method for risk stratification [5]. However, prognostic outcomes can differ significantly among GC patients with the same pathological stage, indicating that this staging system may not fully capture clinical prognosis [6]. Therefore, additional prognostic markers are essential for improving risk stratification and informing personalized treatment decisions.

The albumin–bilirubin (ALBI) grade was developed as a straightforward, objective measure of liver function in patients with hepatocellular carcinoma (HCC) [7]. This scoring system has shown prognostic value not only in HCC but also across various malignancies, reflecting nutritional status, liver reserve function, and overall systemic health [8]. Recent studies have investigated the prognostic utility of ALBI and its modified version (mALBI) in GC, yielding promising results [9, 10]. Moreover, ALBI and mALBI scores have been identified as indicators of chemotherapy tolerance and postoperative complications [11, 12]. The mALBI grade stratifies patients into higher-risk categories, potentially providing superior prognostic stratification compared to the original ALBI classification [13]. Despite the increasing evidence supporting the prognostic value of the mALBI grade in GC, its clinical implementation in metastatic disease remains limited. Furthermore, optimal mALBI grade cutoffs and their applicability across diverse ethnic populations continue to be areas of active investigation.

This study aims to determine the prognostic significance of the mALBI grade in patients with metastatic GC. Specifically, we will evaluate the relationship between mALBI scores and overall survival (OS) and progression-free survival (PFS), as well as identify independent prognostic factors for survival through multivariate analysis.

**Table 1:** Demographic and clinical characteristics of participants

**Variable**	**Valid N**	**Category**	***n* (%) / Mean ± SD**
Age	93	-	64 ± 12
Gender	93	Male	66 (71%)
		Female	27 (29%)
Tumor location	91	Cardia	31 (34.1%)
		Corpus	39 (42.9%)
		Antrum	21 (23.1%)
Vascular invasion	51	No	12 (23.5%)
		Yes	39 (76.5%)
Perineural invasion	45	No	18 (40%)
		Yes	27 (60%)
Lymph node involvement	85	No	21 (24.7%)
		Yes	64 (75.3%)
Distant metastasis	93	Liver	51
		Lung	8
		Bone	10
		Peritoneum	12
		Brain	3
		Other	14
Disease status	93	De novo metastatic	49 (52.7%)
		Recurrent metastatic	44 (47.3%)
HER2 status	66	Negative	50 (75.8%)
		Low	7 (10.6%)
		Positive	9 (13.6%)
ECOG performance status	78	0	25 (32.1%)
		1	28 (35.9%)
		2	17 (21.8%)
		3	8 (10.3%)
Surgery/resection	93	No	54 (58.1%)
		Yes	39 (41.9%)
mALBI grade	93	1	45 (48.4%)
		2a	15 (16.1%)
		2b	21 (22.6%)
		3	12 (12.9%)
Progression	93	No	41 (44.1%)
		Yes	52 (55.9%)
Mortality	93	No	48 (51.6%)
		Yes	45 (48.4%)

Continuous variables are reported as mean ± SD, whereas categorical variables are expressed as *n* (%). Abbreviations: ECOG, Eastern Cooperative Oncology Group; HER2, human epidermal growth factor receptor 2; mALBI, modified albumin-bilirubin; N, number; SD, standard deviation.

## Materials and methods

### Research methodology and participant selection

This retrospective, multicenter cohort study was conducted between August 2021 and June 2025. Inclusion criteria encompassed: individuals aged ≥18 years, histologically confirmed gastric adenocarcinoma with metastases, complete clinical and laboratory data—including serum albumin and total bilirubin levels obtained at the time of metastatic GC diagnosis—and adequate follow-up data (all patients were followed until December 2025 or death) for survival analysis. Exclusion criteria included concurrent hepatobiliary malignancies, severe liver cirrhosis, or other primary hepatic disorders. Patients with missing serum albumin or total bilirubin values at the time of metastatic GC diagnosis were also excluded.

### Data collection

Clinical and demographic information was extracted from medical records, including age, gender, tumor location (cardia, corpus, antrum), lymph node metastasis, distant metastasis sites, presence of vascular and perineural invasion, Eastern Cooperative Oncology Group (ECOG) performance status, human epidermal growth factor receptor 2 (HER2) status, and treatment history. Laboratory parameters included hemoglobin, white blood cell count, neutrophil count, lymphocyte count, platelet count, C-reactive protein, alanine aminotransferase, aspartate aminotransferase, gamma-glutamyl transferase, serum albumin, total and direct bilirubin, arterial lactate, and tumor markers (carcinoembryonic antigen, CA 19-9, CA-125). Information regarding neoadjuvant therapy, surgical intervention, adjuvant therapy, and systemic treatment regimens was also documented.

Given that the cohort included both de novo metastatic patients (those with metastatic disease at diagnosis) and recurrent metastatic patients (those who developed metastases after prior early-stage disease), the metastatic disease diagnosis was selected as a uniform time zero to ensure comparability across the study population and specifically evaluate outcomes related to treatments administered in the metastatic setting. PFS was defined as the duration from the diagnosis of metastatic disease to the occurrence of progression, death without progression, or the final follow-up, whichever occurred first. OS was defined as the interval from the diagnosis of metastatic disease to death or the last follow-up for patients who survived.

Progression was defined based on Response Evaluation Criteria in Solid Tumors (RECIST 1.1). Tumor assessments were performed approximately every 8–12 weeks using computed tomography (CT)/PET-CT, in accordance with routine clinical practice at each participating center. Although this was a retrospective multicenter study, the general approach to progression assessment was consistent across centers, relying on radiological evaluation and/or clinical judgment when imaging was unavailable.

### mALBI score calculation and grading

The ALBI score was calculated using the following formula: (log10 serum total bilirubin (µmol/L) × 0.66) + (serum albumin (g/L) × --0.085) [7].

As laboratory values in our institution are reported in conventional units, bilirubin values were converted from mg/dL to µmol/L by multiplying by 17.1, and albumin values were converted from g/dL to g/L by multiplying by 10 prior to ALBI score calculation.

ALBI grade: ≤ –2.60 indicates grade 1; > –2.60 to ≤ –1.39 indicates grade 2; > –1.39 indicates grade 3. The mALBI grade follows the same grading methodology as the ALBI score but is classified into grades 1, 2a, 2b, and 3, with thresholds of ≤ –2.60 (Grade 1), > –2.60 to ≤ –2.27 (Grade 2a), > –2.27 to ≤ –1.39 (Grade 2b), and > –1.39 (Grade 3) [13]. The mALBI score was calculated at the time of metastatic GC diagnosis.

### Statistical analysis

Continuous variables were presented as medians with interquartile ranges (IQRs). Categorical variables were reported as frequencies and percentages. Fisher’s exact test or the chi-square test was employed to compare categorical variables between low and high mALBI groups. The Mann-Whitney *U* test was utilized for continuous variables. Patients with missing data were excluded from statistical analyses using a “pairwise” method. Consistent with previous studies that have employed binary ALBI groupings in GC [14], the four-grade mALBI classification was retained as the primary analysis, while the binary grouping served as a secondary exploratory analysis to facilitate clinical interpretation.

Survival curves were generated using the Kaplan-Meier method, and intergroup differences were assessed with the log-rank test. Pairwise log-rank comparisons between mALBI grades were performed for descriptive purposes without adjustment for multiple comparisons and should be considered exploratory. The overall log-rank test was employed as the primary inferential test for survival differences across grades. Multivariate Cox proportional hazards regression analysis was conducted to identify independent prognostic factors for OS and PFS. Covariates were selected for multivariate analysis based on clinical relevance and univariate significance. Surgical resection was included to account for potential confounding by indication, as patients who underwent resection may represent a clinically distinct subgroup with better baseline performance status and less advanced disease. ECOG performance status was not included in the multivariate model due to missing data in 15 patients (19.2%), which would have further reduced the already limited evaluable sample size. Results were presented as hazard ratios (HR) accompanied by 95% confidence intervals (CI). Model performance was assessed using both discrimination and calibration. Discrimination was quantified using the concordance statistic (C-index). Internal validation was performed via bootstrap resampling (B = 200 iterations); calibration was evaluated using the optimism-corrected calibration slope implemented in the rms package in R. P-values less than 0.05 were considered statistically significant. Statistical analyses were conducted using Statistical Package for the Social Sciences (SPSS) version 27.0 (IBM Corporation, Armonk, NY, USA). R software version 4.5.2 was used for bootstrap analysis.

### Ethical statement

The study protocol was approved by the Ethics Committee of Antalya City Hospital under decision number 105/2025, dated September 25, 2025. Additional approval was obtained from Van Yüzüncü Yıl University Hospital and Alanya Alaaddin Keykubat University Hospital. The study was conducted in accordance with the principles of the Declaration of Helsinki. Due to the retrospective design of the study and the use of anonymized patient data, informed consent was not required in accordance with the ethics committee approval. Patient confidentiality was maintained throughout the study.

## Results

### Participant characteristics

A total of 114 patients were enrolled in the study between August 2021 and June 2025. Of these, 21 patients were excluded due to missing serum albumin or total bilirubin values at the time of metastatic GC diagnosis; no patients were excluded due to loss to follow-up. As the clinical characteristics of the excluded patients were not documented, the potential for selection bias cannot be fully ruled out. Consequently, a total of 93 patients with metastatic GC were included in this study. The demographic and clinical characteristics of the patients are presented in Table 1.

### mALBI grade distribution and comparison between low and high mALBI groups

The mean ALBI score was --2.38 ± 0.73, with a median of --2.56 (IQR: --2.89 to --1.84). Based on the original mALBI grade classification [13], patients were categorized into four grades: Grade 1 (n = 45, 48.4%), Grade 2a (n = 15, 16.1%), Grade 2b (n = 21, 22.6%), and Grade 3 (n = 12, 12.9%). Utilizing established mALBI grade thresholds, patients were further classified into low mALBI (Grades 1 and 2a, n = 60) and high mALBI (Grades 2b and 3, n = 33) groups, consistent with prior binary grouping approaches in GC [14].

Significant differences were observed between the low and high mALBI groups across various clinical and laboratory parameters (Table 2). The high mALBI group had a significantly greater proportion of males (84.8% vs. 63.3%, *P* ═ 0.029) and a worse ECOG performance status (*P ═* 0.015). Additionally, a higher proportion of patients in the high mALBI group presented with recurrent metastatic disease compared to the low mALBI group (63.6% vs. 38.3%, *P* ═ 0.019). The best response to first-line treatment was comparable between the low and high mALBI groups, with no statistically significant difference (*P ═* 0.969). The levels of the CA 125 tumor marker were significantly elevated in the high mALBI group (median 133.15 vs 20.9 U/mL, *P < * 0.001).

**Table 2:** Comparison of low mALBI and high mALBI

**Variable**	**Category**	**Valid N**	**Low mALBI**		**High mALBI**		***P***	**Effect size (95% CI)**
			**n / Mean** ± **SD**	**% / Med (Q1-Q3)**	**n / Mean** ± **SD**	**% / Med (Q1-Q3)**		
Sex/gender	Male	93	38	63.30%	28	84.80%	**0.029**	**--0.227 (--0.409 –0.039)**
	Female		22	36.70%	5	15.20%		
Tumor location	Cardia	91	21	35.60%	10	31.30%	0.847	
	Corpus		24	40.70%	15	46.90%		
	Antrum		14	23.70%	7	21.90%		
Vascular invasion	No	51	9	33.30%	3	12.50%	0.08	
	Yes		18	66.70%	21	87.50%		
Perineural invasion	No	45	9	36.00%	9	45.00%	0.54	
	Yes		16	64.00%	11	55.00%		
Lymph node involvement	No	85	15	27.80%	6	19.40%	0.386	
	Yes		39	72.20%	25	80.60%		
Disease status	De novo metastatic	93	37	61.70%	12	36.40%	**0.019**	**0.242 (0.05– 0.499)**
	Recurrent metastatic		23	38.30%	21	63.60%		
HER2 status	Negative	66	32	76.20%	18	75.00%	0.802	
	Low		5	11.90%	2	8.30%		
	Positive		5	11.90%	4	16.70%		
ECOG performance status	0	78	19	38.00%	6	21.40%	**0.015**	**0.366 (0.194– 0.588)**
	1		21	42.00%	7	25.00%		
	2		8	16.00%	9	32.10%		
	3		2	4.00%	6	21.40%		
Neoadjuvant therapy	No	93	50	83.30%	26	78.80%	0.587	
	Yes		10	16.70%	7	21.20%		
Surgery/resection	No	93	33	55.00%	21	63.60%	0.419	
	Yes		27	45.00%	12	36.40%		
Adjuvant therapy	No	93	35	58.30%	22	66.70%	0.43	
	Yes		25	41.70%	11	33.30%		
First-line treatment	FOLFOX	83	23	43.40%	15	50.00%	0.413	
	FOLFIRI		2	3.80%	1	3.30%		
	mDCF		3	5.70%	3	10.00%		
	FLOT		11	20.80%	2	6.70%		
	XELOX		0	0.00%	2	6.70%		
	FOLFOX-HERCEPTIN		2	3.80%	1	3.30%		
	FOLFOX-Nivolumab		6	11.30%	2	6.70%		
	Other		6	11.30%	4	13.30%		
			**n / Mean** ± **SD**	**% / Med (Q1-Q3)**	**n / Mean** ± **SD**	**% / Med (Q1-Q3)**		
Second-line treatment	FOLFOX	31	1	5.00%	1	9.10%	0.442	
	FOLFIRI		10	50.00%	4	36.40%		
	mDCF		2	10.00%	0	0.00%		
	FLOT		1	5.00%	0	0.00%		
	XELOX		0	0.00%	1	9.10%		
	FOLFOX-HERCEPTIN		0	0.00%	1	9.10%		
	FOLFOX-Nivolumab		0	0.00%	1	9.10%		
	Carboplatin-paclitaxel		1	5.00%	1	9.10%		
	Other		5	25.00%	2	18.20%		
Diabetes mellitus	No	89	41	70.70%	19	61.30%	0.367	
	Yes		17	29.30%	12	38.70%		
Hypertension	No	91	37	62.70%	22	68.80%	0.565	
	Yes		22	37.30%	10	31.30%		
Coronary artery disease	No	90	47	79.70%	26	83.90%	0.628	
	Yes		12	20.30%	5	16.10%		
Cerebrovascular disease	No	90	57	96.60%	28	90.30%	0.216	
	Yes		2	3.40%	3	9.70%		
Response to first-line treatment	Complete Remission	53	2	5.30%	1	6.70%	0.969	
	Partial Remission		15	39.50%	5	33.30%		
	Stable Disease		8	21.10%	3	20.00%		
	Progression		13	34.20%	6	40.00%		
Progression	No	93	30	50.00%	11	33.30%	0.121	
	Yes		30	50.00%	22	66.70%		
Mortality	No	93	40	66.70%	8	24.20%	**<0.001**	**0.406 (0.199– 0.587)**
	Yes		20	33.30%	25	75.80%		
Age (years)	-	93	63 ± 12	-	66 ± 13	-	0.260	
Tumour size (cm)	-	80	-	5 (3-7)	-	3.8 (2.8-6.8)	0.473	
Haemoglobin (g/dL)	-	93	11.8 ± 2.27	-	10.55 ± 2.05	-	**0.01**	**0.569 (0.134– 0.992)**
WBC count ×10^3^/µL (×10^3^/mm^3^)	-	93	-	8.1 (6.6-10.55)	-	9.2 (5.85-11.42)	0.838	
			**n / Mean** ± **SD**	**% / Med (Q1-Q3)**	**n / Mean** ± **SD**	**% / Med (Q1-Q3)**		
Platelet count ×10^3^/µL (×10^3^/mm^3^)	-	93	291 ± 103	-	348 ± 160	-	0.069	
Lymphocyte count ×10^3^/µL (×10^3^/mm^3^)	-	93	-	1.8 (1.42-2.21)	-	1.44 (1-1.8)	**0.03**	**0.273 (0.051– 0.469)**
Neutrophil count ×10^3^/µL (×10^3^/mm^3^)	-	92	-	5.47 (4-7.54)	-	6.5 (3.78-8.73)	0.46	
Creatinine (mg/dL)	-	93	-	0.88 (0.73-1.01)	-	0.8 (0.63-0.99)	0.166	
ALT (U/L)	-	93	-	13 (10-19)	-	18 (9-33)	0.226	
AST (U/L)	-	91	-	18 (14-26)	-	23 (15-51)	**0.048**	**--0.252 (--0.492 – 0.023)**
ALP (U/L)	-	89	-	79 (62-106)	-	114 (66-274)	0.108	
GGT (U/L)	-	88	-	24 (16-52)	-	56 (21-289)	**0.019**	**--0.306 (0.537– --0.032)**
CA 19-9 (U/mL)	-	84	-	18 (9-164.87)	-	9.88 (3.6-55.55)	0.109	
CEA (ng/mL)	-	87	-	4.06 (1.59-16.35)	-	4.2 (1.81-31)	0.604	
CA 125 (U/mL)	-	58	-	20.9 (11.2-86.7)	-	133.15 (62.05-288)	**<0.001**	**--0.589 (--0.774 – 0.313)**
OS (Days)	-	93	-	561 (298.5-990.5)	-	271 (179-533)	**0.002**	**0,395 (0.157– 0.59)**
PFS (days)	-	93	-	423.5 (224-817.5)	-	199 (98-323)	**<0.001**	**0,453 (0,215– 0,64)**

Patients were classified into two groups according to their mALBI score: Low (Grades 1 and 2a) and high (Grades 2b and 3). Categorical variables are expressed as n (%), while continuous variables are presented as mean ± SD or median (Q1-Q3). *p* values were determined using the chi-square test or Fisher’s exact test for categorical variables, and the Mann-Whitney U test or Student’s t-test for continuous variables. A significance level of *P < * 0.05 was established. Abbreviations: ALP, alkaline phosphatase; ALT, alanine aminotransferase; AST, aspartate aminotransferase; CA 19-9, carbohydrate antigen 19-9; CA 125, cancer antigen 125; CEA, carcinoembryonic antigen; CI, confidence interval; ECOG, Eastern Cooperative Oncology Group; FLOT, fluorouracil, leucovorin, oxaliplatin, and docetaxel; FOLFIRI, folinic acid, fluorouracil, and irinotecan; FOLFOX, folinic acid, fluorouracil, and oxaliplatin; GGT, gamma-glutamyl transferase; HER2, human epidermal growth factor receptor 2; mALBI, modified albumin-bilirubin; mDCF, modified docetaxel, cisplatin, and fluorouracil; Med, median; N, number; OS, overall survival; PFS, progression-free survival; Q1, first quartile; Q3, third quartile; SD, standard deviation; WBC, white blood cell; XELOX, capecitabine and oxaliplatin.

### Survival outcomes

Survival analysis revealed significant differences between the low and high mALBI groups. The median OS was 561 days for the low mALBI group compared to 271 days for the high mALBI group (*P ═* 0.002). Similarly, the median PFS was 423.5 days for the low mALBI group versus 199 days for the high mALBI group (*P < *0.001). The mortality rate was significantly higher in the high mALBI group (75.8% vs. 33.3%, *P < *0.001).

**Figure 1.**
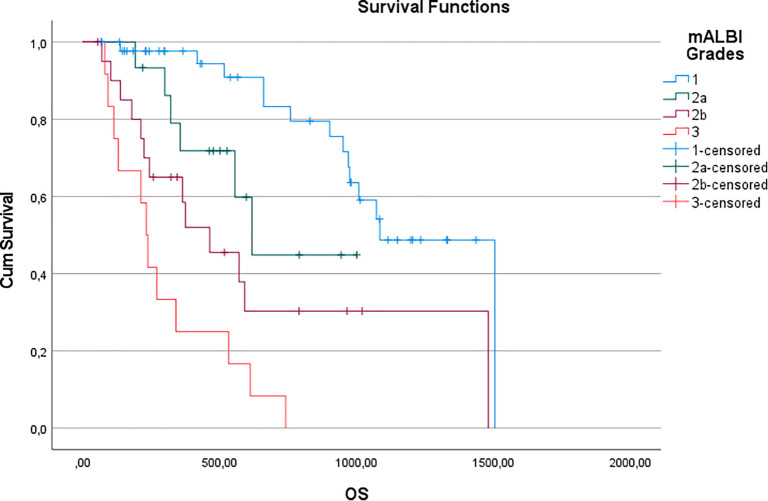
**Overall survival according to modified albumin-bilirubin grade.** Kaplan–Meier curves showing overall survival in patients with metastatic gastric cancer stratified by mALBI grade. Survival decreased progressively with worsening mALBI grade, with Grade 1 showing the most favorable survival profile and Grade 3 showing the poorest survival. The overall difference in overall survival among the four mALBI grades was statistically significant by the log-rank test (χ^2^ ═ 44.156, *P < * 0.001). Pairwise exploratory comparisons showed significantly better overall survival for Grade 1 compared with Grades 2a, 2b, and 3, and poorer survival for Grade 3 compared with Grades 2a and 2b. Censored observations are indicated by tick marks on the curves. Abbreviations: GC, gastric cancer; mALBI, modified albumin-bilirubin; OS, overall survival; χ^2^, chi-square.

Kaplan-Meier survival analysis with log-rank testing demonstrated significant differences in OS across all mALBI grades (χ^2^=44.156, *P < *0.001) (Table 3). Pairwise comparisons indicated that Grade 1 exhibited significantly better OS compared to Grade 2a (*P ═* 0.028), Grade 2b (*P < *0.001), and Grade 3 (*P < *0.001). Grade 2a also showed superior OS compared to Grade 3 (*P ═* 0.001), and Grade 2b was associated with better OS than Grade 3 (*P ═* 0.040) (Figure 1). These exploratory pairwise comparisons should be interpreted with caution, as they were not adjusted for multiple comparisons.

**Table 3:** OS analysis

**Overall comparison (Log rank - Mantel-Cox)**								
**Chi-Square (df)**				***P* value**			
44.156 (3)				<0.001			
**Pairwise comparisons (Log rank - Mantel-Cox)**								
**mALBI Grade**	**1**		**2a**		**2b**		**3**	
	**χ^2^**	***P***	**χ^2^**	***P***	**χ^2^**	***P***	**χ^2^**	***P***
**1**			4.852	0.028	13.152	<0.001	52.556	<0.001
**2a**	4.852	0.028			1.680	0.195	10.737	0.001
**2b**	13.152	<0.001	1.680	0.195			4.215	0.040
**3**	52.556	<0.001	10.737	0.001	4.215	0.040		

The log-rank (Mantel-Cox) test was employed for overall comparisons and for pairwise comparisons between mALBI grades. Chi-square (χ^2^) values, df, and corresponding *p* values are reported, with statistical significance defined as *P < * 0.05. It is important to note that pairwise comparisons are exploratory in nature and have not been adjusted for multiple testing. The primary analysis is the overall comparison conducted using the log-rank test. Abbreviations: Df, degrees of freedom; mALBI, modified albumin-bilirubin; OS, overall survival; χ^2^, chi-square.

**Figure 2.**
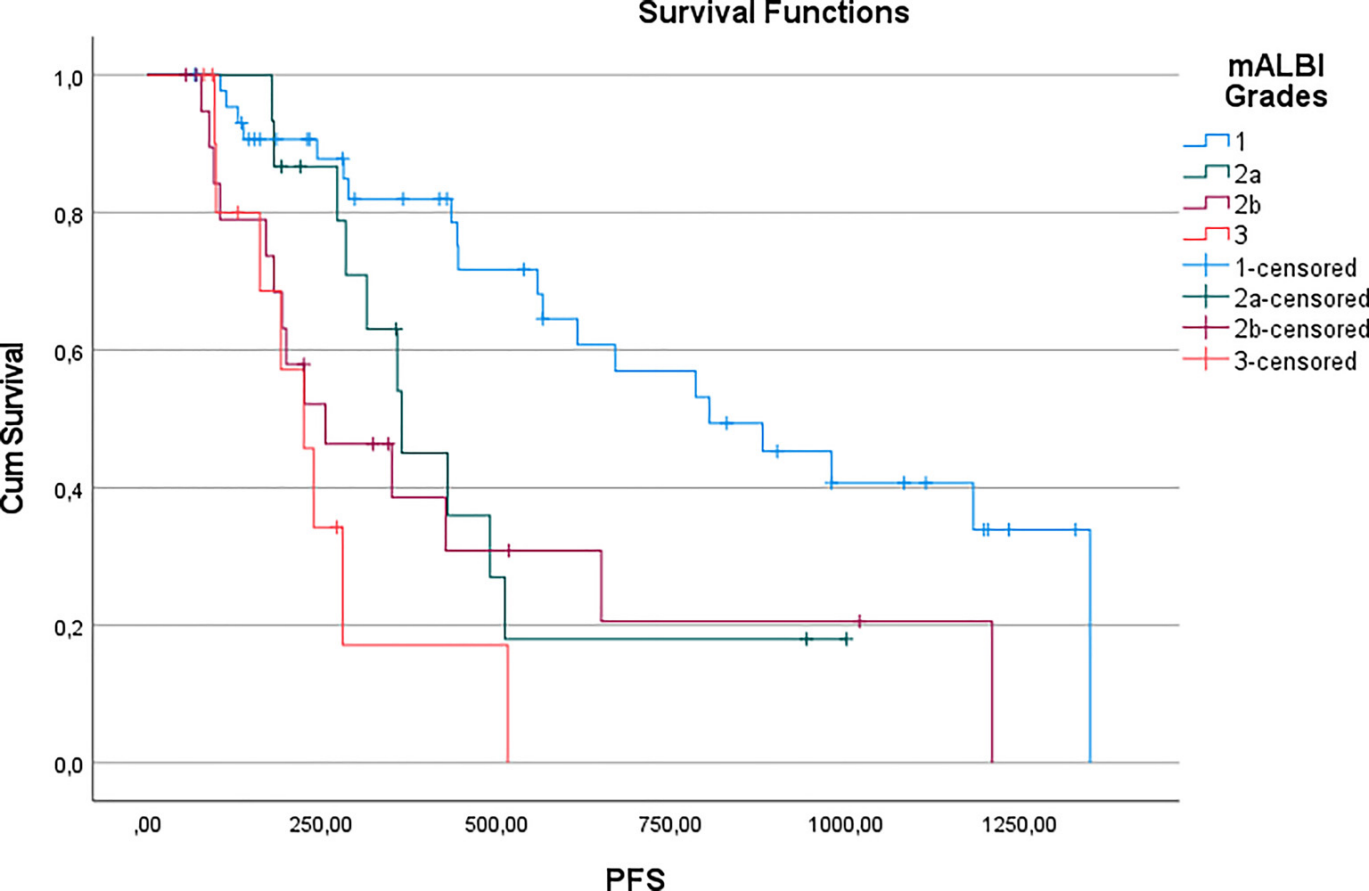
**Progression-free survival according to modified albumin-bilirubin grade.** Kaplan–Meier curves showing progression-free survival in patients with metastatic gastric cancer stratified by modified albumin-bilirubin (mALBI) grade. Progression-free survival declined progressively with increasing mALBI grade, with Grade 1 demonstrating the longest progression-free survival and Grade 3 the shortest. The overall difference among the four mALBI grades was statistically significant by the log-rank test (χ^2^ ═ 22.142, *P < * 0.001). Exploratory pairwise comparisons showed significantly longer progression-free survival for Grade 1 compared with Grades 2a, 2b, and 3, and for Grade 2a compared with Grade 3. Censored observations are indicated by tick marks on the curves. Abbreviations: GC, gastric cancer; mALBI, modified albumin-bilirubin; PFS, progression-free survival; χ^2^, chi-square.

For PFS, the overall comparison among mALBI grades was highly significant (χ^2^ = 22.142, *P < *0.001) (Table 4). Pairwise comparisons revealed that Grade 1 had significantly longer PFS than Grade 2a (*P ═* 0.023), Grade 2b (*P ═* 0.002), and Grade 3 (*P < *0.001). Grade 2a demonstrated better PFS compared to Grade 3 (*P ═* 0.036) (Figure 2). These exploratory pairwise comparisons should be interpreted with caution, as they were not adjusted for multiple comparisons.

**Table 4:** PFS analysis

**Overall comparison (Log rank - Mantel-Cox)**								
**Chi-Square (df)**				***P* value**			
22.142 (3)				<0.001			
**Pairwise comparisons (Log rank - Mantel-Cox)**								
**mALBI Grade**	**1**		**2a**		**2b**		**3**	
	**χ^2^**	***P***	**χ^2^**	***P***	**χ^2^**	***P***	**χ^2^**	***P***
**1**			5.206	**0.023**	9.577	**0.002**	22.141	**<0.001**
**2a**	5.206	**0.023**			0.490	0.484	4.389	**0.036**
**2b**	9.577	**0.002**	0.490	0.484			1.118	0.290
**3**	22.141	**<0.001**	4.389	**0.036**	1.118	0.290		

The log-rank (Mantel-Cox) test was employed for overall comparisons as well as pairwise comparisons among mALBI grades. The results include chi-square (χ^2^) values, df, and corresponding *p* values, with statistical significance defined as *P < * 0.05. It is important to note that pairwise comparisons are exploratory and have not been adjusted for multiple testing. The overall comparison using the log-rank test serves as the primary analysis. Abbreviations: Df, degrees of freedom; mALBI, modified albumin-bilirubin; PFS, progression-free survival; χ^2^, chi-square.

### Multivariate Cox regression analysis

Multivariate Cox regression analysis was performed to identify independent prognostic factors for OS and PFS (Table 5). The variables included in the model were not clinically related. The variance inflation factor (VIF) analysis revealed that the variables did not demonstrate multicollinearity, with a maximum VIF (generalized variance inflation factor, GVIF) of 1.232. The Cox regression model included 93 patients; however, 91 were evaluable after SPSS automatically excluded 2 patients who were censored before the earliest event in a stratum. Among evaluable patients, 45 experienced death, resulting in an events-per-variable ratio of 7.5. The mALBI grade emerged as a statistically significant independent predictor for both OS (*P < *0.001) and PFS (*P ═* 0.001). Compared to Grade 1 (reference category), Grade 2a was associated with a 2.1-fold increase in mortality risk; however, this did not reach statistical significance (HR=2.129, 95% CI: 0.768–5.901, *P ═* 0.146). Grade 2b demonstrated a 5.4-fold increase in mortality risk relative to Grade 1 (HR=5.399, 95% CI: 2.320–12.565, *P < *0.001). Furthermore, Grade 3 was associated with a markedly elevated mortality risk, exhibiting an approximately 10.7-fold increase compared to the reference group (HR=10.695, 95% CI: 4.321–26.467, *P < *0.001).

**Table 5:** Univariate and multivariate Cox regression analyses for OS and PFS

**Overall survival**								
	**Univariate analysis**				**Multivariate analysis**			
**Variables**	**HR**	**% 95 CI**		***p* value**	**HR**	**% 95 CI**		***p* value**
**Age**	1.035	1.006	1.065	0.017	1.044	1.011	1.078	0.008
**No surgery (Ref. surgery)**	2.502	1.267	4.941	0.008	2.499	1.163	5.367	0.019
**Disease status (Ref. DeNovo)**	1.961	1.020	3.774	0.044	1.034	0.520	2.055	0.924
**mALBI grade (Ref. mALBI grade 1)**				<0.001				<0.001
**mALBI grade 2a**	2.440	0.890	6.684	0.083	2.129	0.768	5.901	0.146
**mALBI grade 2b**	4.020	1.816	8.897	<0.001	5.399	2.320	12.565	<0.001
**mALBI grade 3**	11.999	5.051	28.504	<0.001	10.695	4.321	26.467	<0.001
**Progression free survival**								
	**Univariate analysis**				**Multivariate analysis**			
**Variables**	**HR**	**% 95 CI**		***p* value**	**HR**	**% 95 CI**		***p* value**
**Age**	1.013	0.988	1.039	0.323	1.015	0.986	1.045	0.316
**No surgery (Ref. surgery)**	2.071	1.150	3.729	0.015	2.011	1.053	3.839	0.034
**Disease status (Ref. DeNovo)**	2.280	1.270	4.091	0.006	1.619	0.876	2.993	0.124
**mALBI grade (Ref. mALBI grade 1)**				<0.001				0.001
**mALBI grade 2a**	2.294	1.049	5.015	0.038	1.834	0.827	4.067	0.136
**mALBI grade 2b**	3.005	1.494	6.043	0.002	3.560	1.714	7.393	<0.001
**mALBI grade 3**	6.617	2.558	14.920	<0.001	4.264	1.701	10.687	0.002

Variables assessed include the mALBI grade (with Grade 1 as the reference category, not shown in the table), age, surgical resection status, and disease status (de novo versus recurrent metastatic). Results are presented as HRs, 95% CIs, and *p* values, with statistical significance established at *P < * 0.05. Reference Categories: MALBI: Grade 1; Surgical Resection: Yes; Disease Status: De Novo Metastatic; For the PFS analysis, four patients were excluded due to censoring prior to the occurrence of the earliest event in a stratum, resulting in an evaluable population of 89 patients. Abbreviations: CI, confidence interval; HR, hazard ratio; mALBI, modified albumin-bilirubin; OS, overall survival; PFS, progression-free survival; Ref, reference.

The Cox regression model included 93 patients, of whom 89 were evaluable after SPSS automatically excluded 4 patients who were censored before the earliest event in a stratum. Among evaluable patients, 52 experienced progression, yielding an events-per-variable ratio of 8.6. Compared to Grade 1 (reference category), Grade 2a was associated with a 1.8-fold increase in progression risk; however, this association did not reach statistical significance (HR=1.834, 95% CI: 0.827–4.067, *P ═* 0.136). Grade 2b demonstrated a 3.5-fold increase in progression risk relative to Grade 1 (HR = 3.560, 95% CI: 1.714–7.393, *P < *0.001). Furthermore, Grade 3 was associated with an elevated progression risk, with a 4.2-fold increase compared to the reference group (HR=4.264, 95% CI: 1.701–10.687, *P ═* 0.002).

To assess model stability given the limited events-per-variable ratio, a bootstrap resampling procedure with 200 iterations was performed to estimate the optimism-corrected C-index for the multivariate Cox model. The discriminative ability of the multivariate Cox model was evaluated using the C-index, calculated in R (version 4.5.2). Bootstrap internal validation indicated good discrimination for the OS model (apparent C-index = 0.809; bootstrap-corrected C-index = 0.820) and acceptable discrimination for the PFS model (apparent C-index = 0.720; bootstrap-corrected C-index = 0.733). Calibration slopes after bootstrap correction were 0.809 for OS and 0.754 for PFS, indicating modest optimism consistent with the limited events-per-variable ratio.

## Discussion

Our retrospective findings demonstrate a clear gradient in survival outcomes across mALBI grades, with low mALBI grades (1 and 2a) exhibiting a significantly better prognosis than higher grades. Furthermore, mALBI grade was independently associated with both OS and PFS in patients with metastatic GC. These results suggest that baseline liver function, as assessed by the mALBI score, may significantly influence treatment outcomes and prognosis in metastatic GC.

The mALBI grading system utilizes only two objective laboratory parameters—serum albumin and total bilirubin—rendering it more practical and reproducible than traditional liver function classification systems such as the Child-Pugh score [15]. Recent studies have established the prognostic significance of ALBI and mALBI scores in GC [10, 12, 14, 16]. Our research extends these findings specifically to the metastatic GC population, demonstrating robust prognostic discrimination across all mALBI grades. The biological rationale for employing the mALBI grade in GC prognosis is multifaceted. Hypoalbuminemia indicates both malnutrition and the acute-phase response linked to cancer cachexia and systemic inflammation. Low albumin levels consistently correlate with poor outcomes in various cancers, including GC [17, 18]. Elevated bilirubin levels, although often subtle in the absence of overt liver metastases, may signify subclinical liver dysfunction, biliary obstruction, or increased tumor burden affecting hepatic metabolism [19]. Given that most metastases in our study were liver metastases, we posit that a scoring system based on albumin and bilirubin levels, which reflect liver reserve, serves as a significant prognostic indicator.

Zhu et al. demonstrated that high preoperative ALBI levels are an independent risk factor for survival in advanced GC; however, all patients in that study underwent radical resection surgery, with a majority categorized in the high ALBI group [20]. In contrast, our study cohort primarily consisted of patients with metastatic disease, most of whom had mALBI grade 1. This can be attributed to the fact that, at diagnosis, patients’ nutritional and liver reserves had not yet been compromised. Shoka et al. indicated that mALBI grade 3 (patients categorized into grades 1, 2, and 3) was an independent prognostic factor for disease-specific OS in preoperative GC patients [10]. Our study stratified patients into four groups (grades 1, 2a, 2b, and 3) according to the original classification [13].

A recent systematic review and meta-analysis reported that a higher ALBI grade correlates with worse OS and PFS, consistent with our findings [16]. In Wang et al.’s study [14], high preoperative ALBI scores and TNM stage were identified as independent risk factors for survival in patients with stage I-III GC. Given that all patients in our study were metastatic (stage IV), we underscored the prognostic importance of the mALBI score within this patient group. Although all participants had metastatic disease, detailed TNM staging data, including tumor burden and disease distribution within stage IV, were not available for all patients and could not be included in the multivariate model. This limitation is significant, as these factors may independently influence prognosis even in a metastatic context.

We observed that patients with high mALBI scores exhibited significantly elevated CA-125 levels. While CA 125 is traditionally linked to ovarian cancer, it has been shown to be elevated in GC, particularly in cases with peritoneal involvement [21]. The correlation between high mALBI and elevated CA 125 may reflect increased peritoneal disease burden and associated ascites formation, both of which contribute to hypoalbuminemia and poor prognosis.

The association between high mALBI and inferior ECOG performance status highlights the interplay between liver function, nutritional status, and functional capacity. Patients with compromised liver function and lower albumin levels are more likely to experience fatigue, weakness, and decreased chemotherapy tolerance, all of which contribute to worse performance status and ultimately inferior survival outcomes [22].

Interestingly, we found no statistically significant difference in the rate of GC progression between patients in the high and low mALBI groups (*P ═* 0.121). In contrast, mortality rates were significantly different between these groups (*P < *0.001). Since the mALBI score reflects nutritional status, higher mortality in the high mALBI group may be attributed to poor nutritional status, despite similar disease progression compared to the low mALBI group.

In our multivariate analysis, the absence of prior surgical resection was associated with worse OS and PFS; however, this finding should be interpreted with caution. Due to the unavailability of data on surgical intent, timing, and extent (radical vs. palliative), this association may reflect confounding by indication rather than a true independent prognostic effect. Patients who did not undergo surgery may have presented with more advanced disease or poorer baseline performance status, rendering them less likely surgical candidates.

Previous studies in GC have highlighted the prognostic value of various inflammatory and nutritional markers, including the neutrophil-to-lymphocyte ratio, prognostic nutritional index, platelet-to-lymphocyte ratio, and Glasgow Prognostic Score [23–25]. The mALBI score offers several advantages over these markers: it is derived from routine laboratory tests, is objective and reproducible, and has been validated across multiple cancer types [26]. Our study confirmed that the mALBI score retains its prognostic value even in the presence of other established prognostic factors such as tumor characteristics, age, and surgical resection status. However, previous research has indicated that the instability and individual variability of blood biochemical markers may limit their prognostic utility in GC patients [27].

This multicenter study, which exclusively included patients with metastatic GC, underscores the clinical implications of our findings. The mALBI score may have potential for integration into clinical practice to aid in patient stratification; however, prospective validation is necessary prior to routine clinical implementation. Patients with elevated mALBI scores could benefit from more intensive supportive care, nutritional interventions, and potentially modified chemotherapy regimens.

This study has certain limitations that warrant consideration. The retrospective design may restrict generalizability. The sample size for mALBI Grade 3 (n ═ 12) and Grade 2b (n ═ 21) was relatively small, potentially reducing statistical power and resulting in imprecise hazard ratio estimates with wide confidence intervals, particularly for higher mALBI grades. These estimates should be interpreted cautiously, and replication in larger cohorts is warranted. Additionally, we did not assess changes in mALBI scores over time or in response to treatment, which could provide further prognostic insights. The absence of clinical staging and tumor differentiation data constitutes another significant limitation. Bootstrap-corrected calibration slopes of 0.809 (OS) and 0.754 (PFS) suggest modest overfitting, which is anticipated given the limited sample size and events-per-variable ratio. While internal validity was analyzed, external validation in an independent cohort is required to confirm the generalizability of these models. Information regarding types of surgical treatment (radical vs. palliative) was unavailable. Although surgical resection was included in the multivariate model to control for confounding by indication, residual confounding cannot be fully excluded due to the retrospective design and lack of detailed surgical candidacy criteria. ECOG performance status, which varied significantly between low and high mALBI groups, could not be included in the multivariate model due to missing data in 15 patients, leaving open the possibility of residual confounding. Finally, treatment regimen heterogeneity may have influenced survival outcomes.

## Conclusion

Patients with lower mALBI grades exhibited significantly better survival outcomes than those with higher grades. Given its straightforward calculation from routine laboratory tests, the mALBI grade may serve as a valuable prognostic marker in metastatic GC; however, prospective validation in larger and more diverse cohorts is essential before its incorporation into standard clinical practice. Further prospective studies with larger sample sizes are needed to validate these findings and ascertain whether interventions aimed at improving nutritional status and liver function can enhance outcomes in high-risk patients.

## Data Availability

The datasets used and/or analysed during the current study are available from the corresponding author on reasonable request.

## References

[1] Sung H, Ferlay J, Siegel RL, Laversanne M, Soerjomataram I, Jemal A (2021). Global cancer statistics 2020: GLOBOCAN estimates of incidence and mortality worldwide for 36 cancers in 185 countries. CA Cancer J Clin..

[2] Lei ZN, Teng QX, Tian Q, Chen W, Xie Y, Wu K (2022). Signalling pathways and therapeutic interventions in gastric cancer. Signal Transduct Target Ther..

[3] Yang WJ, Zhao HP, Yu Y, Wang JH, Guo L, Liu JY (2023). Updates on global epidemiology, risk and prognostic factors of gastric cancer. World J Gastroenterol..

[4] Burz C, Pop V, Silaghi C, Lupan I, Samasca G (2024). Prognosis and treatment of gastric cancer: a 2024 update. Cancers (Basel)..

[5] Amin MB, Greene FL, Edge SB, Compton CC, Gershenwald JE, Brookland RK (2017). The eighth edition AJCC Cancer Staging Manual: continuing to build a bridge from a population-based to a more “personalized” approach to cancer staging. CA Cancer J Clin..

[6] Yun JH, Choi YY, Cheong JH (2025). The current evidence and future direction of adjuvant treatment for gastric cancer in the era of precision medicine. Cancer Res Treat..

[7] Johnson PJ, Berhane S, Kagebayashi C, Satomura S, Teng M, Reeves HL (2015). Assessment of liver function in patients with hepatocellular carcinoma: a new evidence-based approach—the ALBI grade. J Clin Oncol..

[8] Hsu WF, Hsu SC, Chen TH, Lin CH, Lin YC, Chang YW (2022). Modified albumin-bilirubin model for stratifying survival in patients with hepatocellular carcinoma receiving anticancer therapy. Cancers (Basel)..

[9] Kanda M, Tanaka C, Kobayashi D, Uda H, Inaoka K, Tanaka Y (2018). Preoperative albumin-bilirubin grade predicts recurrences after radical gastrectomy in patients with pT2–4 gastric cancer. World J Surg..

[10] Shoka M, Kanda M, Ito S, Mochizuki Y, Teramoto H, Ishigure K (2023). Modified albumin-bilirubin grade optimized for risk stratification of patients with stage II–III gastric cancer. Surg Today..

[11] Miwa T, Kanda M, Tanaka C, Kobayashi D, Hayashi M, Yamada S (2019). Albumin-bilirubin score predicts tolerability to adjuvant S-1 monotherapy after curative gastrectomy. J Gastric Cancer..

[12] Hou S, Yu Y, Li N, Yu W, Dai Z, Li H (2025). Preoperative albumin-bilirubin grade combined with sarcopenia predicts long-term outcomes after laparoscopic gastrectomy for advanced gastric cancer. BMC Gastroenterol..

[13] Hiraoka A, Kumada T, Tsuji K, Takaguchi K, Itobayashi E, Kariyama K (2019). Validation of modified ALBI grade for more detailed assessment of hepatic function in hepatocellular carcinoma patients: a multicenter analysis. Liver Cancer..

[14] Wang X, Zheng J, Yang H, Yang X, Cai W, Chen X (2023). Prognostic value of the preoperative albumin-bilirubin score among patients with stages I–III gastric cancer undergoing radical resection: a retrospective study. Clin Transl Sci..

[15] Pugh RNH, Murray-Lyon IM, Dawson JL, Pietroni MC, Williams R (1973). Transection of the oesophagus for bleeding oesophageal varices. Br J Surg..

[16] Omouri-Kharashtomi M, Ghoshouni H, Ayati Firoozabadi A, Mahdizadeh F, Anushiravani A (2026). Prognostic value of albumin-bilirubin (ALBI) grade in patients with gastric cancer: a systematic review and meta-analysis. J Gastrointest Cancer..

[17] Oñate-Ocaña LF, Aiello-Crocifoglio V, Gallardo-Rincón D, Herrera-Goepfert R, Brom-Valladares R, Carrillo JF (2007). Serum albumin is a significant prognostic factor for patients with gastric carcinoma. Ann Surg Oncol..

[18] Liu X, Sun X, Liu J, Kong P, Chen S, Zhan Y (2015). Preoperative C-reactive protein/albumin ratio predicts prognosis of patients after curative resection for gastric cancer. Transl Oncol..

[19] Carr BI, Pancoska P, Branch RA (2009). Tumor and liver determinants of prognosis in unresectable hepatocellular carcinoma: a large case cohort study. Hepatol Int..

[20] Zhu C, Wang X, Chen S, Yang X, Sun J, Pan B (2020). Efficacy of the preoperative albumin-bilirubin grade for predicting survival and outcomes of postoperative chemotherapy for advanced gastric cancer. Cancer Manag Res..

[21] Emoto S, Ishigami H, Yamashita H, Yamaguchi H, Kaisaki S, Kitayama J (2012). Clinical significance of CA125 and CA72-4 in gastric cancer with peritoneal dissemination. Gastric Cancer..

[22] Buccheri G, Ferrigno D, Tamburini M (1996). Karnofsky and ECOG performance status scoring in lung cancer: a prospective, longitudinal study of 536 patients from a single institution. Eur J Cancer..

[23] Deng H, He Y, Huang G, Huang Y, Wu J, Qin X (2024). Predictive value of prognostic nutritional index in patients undergoing gastrectomy for gastric cancer: a systematic review and meta-analysis. Medicine (Baltimore)..

[24] Tan S, Zheng Q, Zhang W, Zhou M, Xia C, Feng W (2024). Prognostic value of inflammatory markers NLR, PLR, and LMR in gastric cancer patients treated with immune checkpoint inhibitors: a meta-analysis and systematic review. Front Immunol..

[25] Shimoda Y, Fujikawa H, Komori K, Watanabe H, Kano K, Yamada T (2022). Preoperative utility of the Glasgow prognostic score on outcomes of patients with locally advanced gastric cancer. J Gastrointest Cancer..

[26] Toyoda H, Johnson PJ (2022). The ALBI score: from liver function in patients with HCC to a general measure of liver function. JHEP Rep..

[27] Shao J, Jiang Z, Jiang H, Ye Q, Jiang Y, Zhang W (2024). Machine learning radiomics liver function model for prognostic prediction after radical resection of advanced gastric cancer: a retrospective study. Ann Surg Oncol..

